# Microplastic abundance in beach sediments of the Kiel Fjord, Western Baltic Sea

**DOI:** 10.1007/s11356-020-12220-x

**Published:** 2021-01-23

**Authors:** Kevin Schröder, Elke Kossel, Mark Lenz

**Affiliations:** 1 Faculty of Agricultural and Nutritional Sciences, University of Kiel, Olshausenstr. 40, 24098 Kiel, Germany; 2 Marine Biogeochemistry, GEOMAR Helmholtz Centre for Ocean Research Kiel, Wischhofstr. 1 – 3, 24148 Kiel, Germany; 3 Marine Ecology, GEOMAR Helmholtz Centre for Ocean Research Kiel, Düsternbrooker Weg 20, 24105 Kiel, Germany

**Keywords:** Microplastics, Beach sediment, Western Baltic Sea, Raman spectroscopy, Beach use, Sewage plant, Grain size, Kiel Fjord

## Abstract

We assessed the abundance of microplastics (0.2–5 mm) in drift line sediments from three sites in Kiel Fjord, Western Baltic Sea. The first site is intensively used by beach visitors, the second is in close proximity to a sewage plant and the third is polluted with large-sized plastic litter. Samples were split into three grain size classes (0.2–0.5, 0.5–1, 1–5 mm), washed with calcium chloride solution, and filtered at 0.2 mm. Filters were then visually inspected, and a total of 180 fragments was classified as microplastics, of which 39% were analyzed using Raman spectroscopy. At the site that is close to a sewage plant as well as at the site with intense beach use, 1.8 and 4.5 particles (fibers plus fragments) per kg of dry sediment were found, respectively, while particle abundances reached 30.2 per kg of dry sediment at the site with high litter loads. Our data suggest that the fragmentation of large plastic debris at site seems to be a relevant source for microplastics in Western Baltic Sea beach sediments.

**Supplementary Information:**

The online version contains supplementary material available at 10.1007/s11356-020-12220-x.

## Introduction

The global production of plastics has increased from 1.7 million tons in 1950 (PlasticsEurope 2012) to 359 million tons in 2018 (PlasticsEurope 2019). The material has become omnipresent in daily life, e.g., as packaging or construction materials or in clothes, and it became a substantial part of our garbage. After several decades of plastic litter release into the marine environment, the negative effects of this pollution on marine wildlife, e.g., through ingestion, entanglement, and intoxication, have been described by studies on various taxonomic groups (Gall and Thompson 2015; Worm et al. 2017). Furthermore, the potential trophic transfer and bioaccumulation of plastic through the marine food web could ultimately also affect human consumers (Worm et al. 2017). Estimations suggest that between 4.8 and 12.7 million tons of plastic litter entered the oceans in 2010 (Jambeck et al. 2015), while 60% of this load are supposed to have reached the open ocean due to the influence of surface currents and winds (Maximenko et al. 2012; Lebreton et al. 2012). The remaining share is deposited along the coastlines of continents and islands, where a presumably large but still not quantified part of the debris gets fragmented by photolysis, thermal oxidation, hydrolysis, biodegradation, or mechanical fragmentation. By this, it turns into secondary microplastics (Browne 2015; Dümichen et al. 2017).

In addition to this, primary microplastics pollute the marine environment, which are, for example, beads that serve as scrubbers in cosmetics or in sandblasting, or pre-production pellets (Andrady 2011; Fendall and Sewell 2009).

Microplastics in the marine environment have been found at the sea surface, in the water column, in sediments, and even in sea ice (Thompson 2015). Sediments are presumably the ultimate sink for microplastics, since also particles that initially have positive buoyancy get colonized by bacteria and eukaryotes or they get embedded in fecal pellets and, as a consequence, can sink to the sea floor (Cole et al. 2016). Sediments that contain microplastics were found at beaches in the sub- as well as in the intertidal, in rivers, in estuaries, and in the deep sea (Hanvey et al. 2017). However, previous findings are difficult to compare as they are based on different sampling, extraction, and identification techniques and also considered different particle size ranges. Furthermore, the quantification of the abundance of microplastics was not uniform and the studies most often lack temporal or spatial resolution. Due to these shortcomings, we still have a substantial lack of information about the pollution of our coasts and seas with microplastics (Hanvey et al. 2017). This also applies to the Baltic Sea.

In this sea area, studies on particle concentrations in the water column or at the water surface were carried out in the main Baltic Proper basins (Bagaev et al. 2018; Beer et al. 2018), in Denmark (Tamminga et al. 2018), in Sweden (Gewert et al. 2017; Schönlau et al. 2020), in Russia (Zobkov et al. 2019), in Germany (Ory et al. 2020), and in the Gulf of Finland (Ojaveer et al. 2013; Talvite et al. 2015; Setälä et al. 2016). Seafloor or beach sediments were investigated in Finland (Talvite et al. 2015; Näkki et al. 2019), Russia (Zobkov and Esiukova 2017; Esiukova 2017; Chubarenko et al. 2018; Esiukova et al. 2020), Lithuania (Chubarenko et al. 2020; Esiukova et al. 2020), and Germany (Stolte et al. 2015; Hengstmann et al. 2018) as well as along the Polish coast (Graca et al. 2017; Urban-Malinga et al. 2020). Due to their dynamic character, beach sediments could not only serve as a sink (Chubarenko et al. 2018) but also as a source (Critchell and Lambrechts 2016) for microplastics in coastal seas and, because of their large spatial extension, can store substantial amounts of small-sized plastic debris. Van Cauwenberghe et al. (2013), for example, calculated that a load of 13 micro-sized particles per kilogram of dry sediment corresponds to a total particle load of 2.1 × 10^7^ along a beach of 100 m in length and 250 m in width, even when considering only the upper 5 cm of the sediment layer (Van Cauwenberghe et al. 2013).

Beaches are an interface at which humans come in direct contact with marine microplastics. As microplastics may also have negative impacts on human health (Prata et al. 2020), the interest of beach users, tourists, and tourism managers, in particular in the southwest Baltic Sea region, in this type of environmental pollution is generally high. Furthermore, according to the European Marine Strategy Framework Directive, marine litter (which also comprises microplastics) is a descriptor for the ecological status of water bodies (EU 2008; EU 2010). Thus, assessing the abundance of microplastics in coastal environments is of importance for environmental managers. Additionally, the southwest Baltic Sea including the Kiel Fjord comprises important spawning grounds for ecologically and economically important fish species (Hüssy 2011; Polte et al. 2013). Microplastics in beach sediments, which are a part of the sea-land interface, can therefore potentially also affect marine species. We provide first data from an embayment in the Western Baltic Sea that is heavily used for water sports and tourism and that experiences intense ship traffic.

## Materials and methods

### Sampling sites

**Fig. 1:**
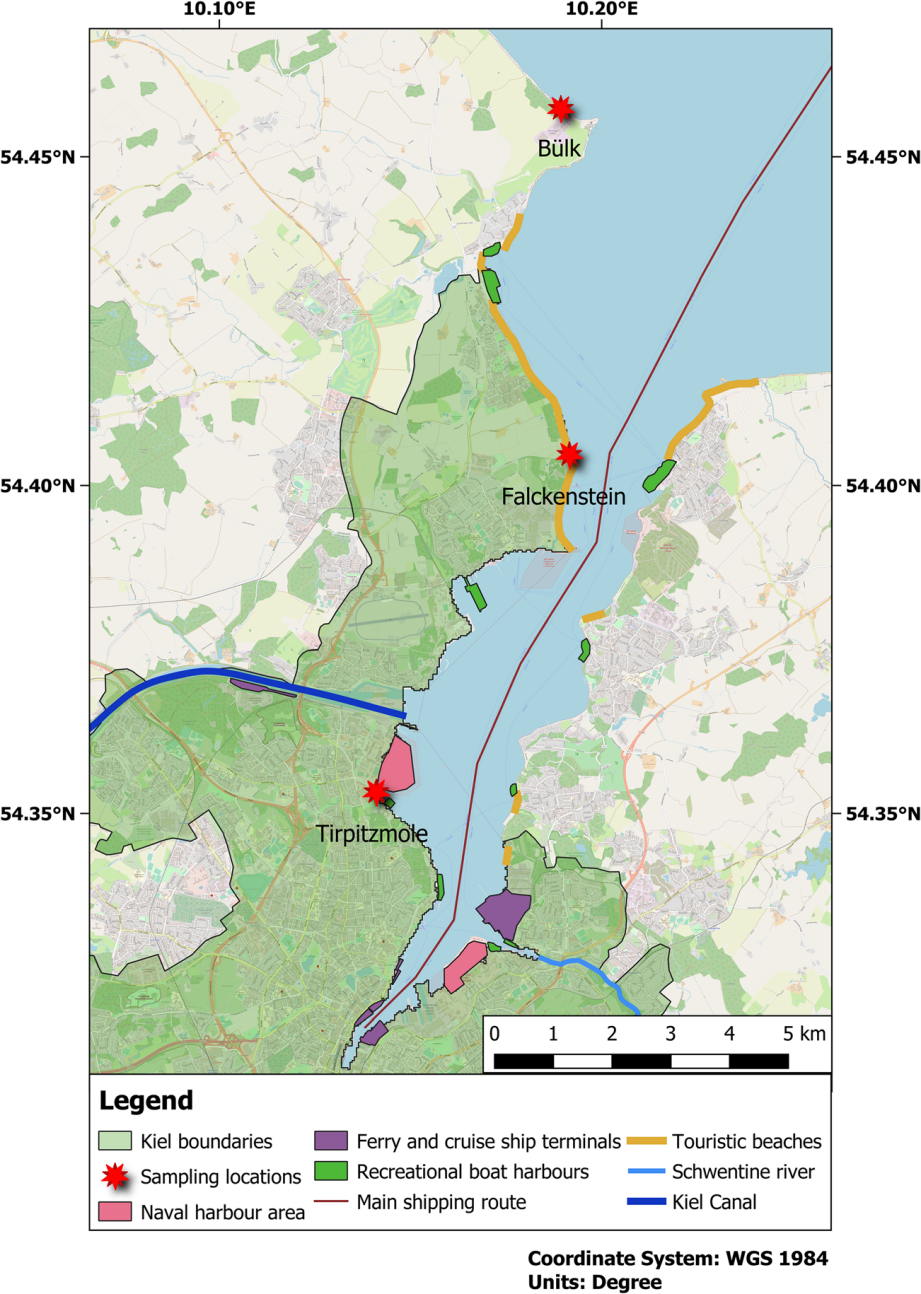
Locations of the three sampling sites along the western shore of the Kiel Fjord, Western Baltic Sea. Potential sources for microplastics such as harbors, terminals, beaches, and waterways within Kiel Fjord are displayed. The green shaded area represents the city area of Kiel (© OpenStreetMap contributors 2018)

Three sites at a distance of ~ 6 km to each other were sampled along the western shore of the Kiel Fjord, Western Baltic Sea, between September 24 and October 3, 2016, to inspect drift line sediments for the presence of microplastics (Fig. 1). Kiel Fjord is a mesohaline inner coastal water body with salinities ranging from 2.6 to 22.4 with a mean of 14.3. Temporal variability in salinity originates from the influx of saline waters from the North Sea and of brackish waters from the eastern Baltic Sea. The Kiel Fjord is, at its mouth, about 6 km wide, while it is 15 km long. Its average water depth is 10 m (Schröder et al. 2014). At approximately half of its length, the Schwentine River with a catchment area of 722.55 km^2^ and an annual mean discharge of 202 million m^3^ flows into the fjord, which has a total shoreline length of approximately 34 km (Landesbetrieb für Küstenschutz, Nationalpark und Meeresschutz Schleswig-Holstein 2015). The city of Kiel with 249,000 inhabitants is surrounding the fjord along 26.5 km of its shoreline, while the other shore areas are occupied by beaches, which are frequently used by locals and by tourists. Furthermore, they accommodate marinas, industrial sites, shipyards, and naval harbors.

**Fig. 2:**
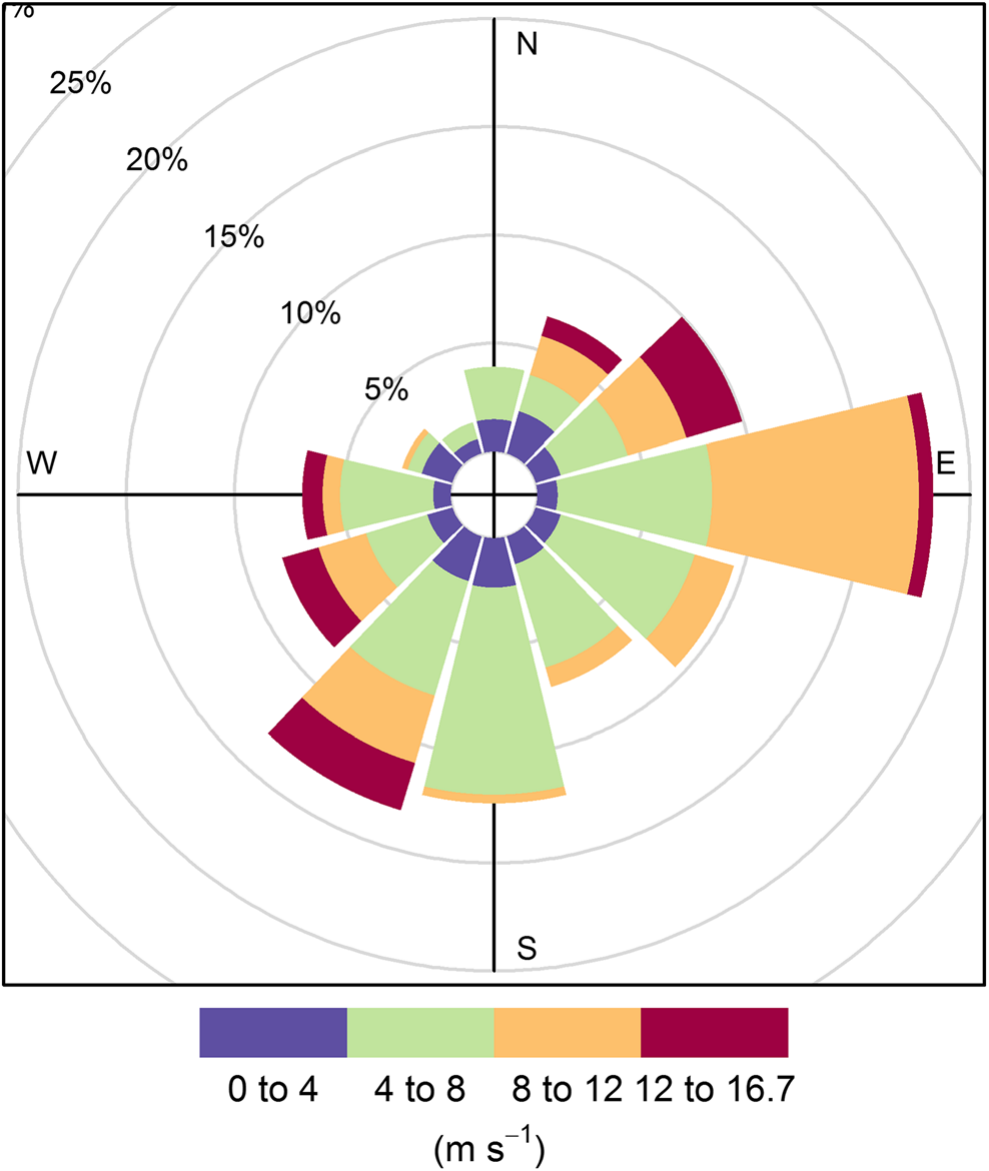
Wind rose of Kiel Lighthouse at 54.50° N; 10.27° E during the period from September 3 to October 4, 2016. Wind direction is binned in classes of 30°

The most northern of the three sampling sites was located near the outlet of the Bülk sewage treatment plant (54° 27′ 26.48″ N; 10° 11′ 20.33″ E). The plant is treating waste waters from 370,000 households in Kiel and its vicinities, which all together have an annual volume of approximately 19 million m^3^. The cleared waters are released into the Baltic Sea by a pipeline, which is 1040 m in length and which leads perpendicularly away from the shore. The Y-shaped end of the pipeline is facing northwards. The second sampling site was located in Falckenstein (54° 24′ 14.68″ N; 10° 11′ 32.05″ E), which is a popular, east-ward facing beach destination in the outer part of the Kiel Fjord. The third and most southern sampling site was at the east-ward facing Tirpitzmole (54° 21′ 11.47″ N; 10° 8′ 27.84″ E), where a short strip of sandy sediment is located between a naval base and a marina. Because of its protected position, a large amount of flotsam accumulates here (Kevin Schröder, personal observation). The predominant wind direction in the Kiel Fjord area during the sampling period (September to October 2016) is shown in Fig. 2.

### Sediment sampling

Sediment samples were collected with a metal shovel covering a quadratic area of approximately 0.4 m^2^ down to a depth of 4–6 cm, since more than 50% of the present microplastics are usually found in the upper 5 cm of the sediment (Carson et al. 2011). Furthermore, samples were taken at the drift line, i.e., the area close to the water line where debris accumulates. Since the Baltic Sea exhibits a tidal range of only 20 cm, fluctuations in the water level and in the location of the drift line along the shores of Kiel Fjord are mainly wind-driven (Healey et al. 2002). The average water level amplitude in the fjord during the course of a year is 2.45 m (WSV 2018), while during the sampling period in 2016 the water level fluctuated by 1.20 m.

During sampling, short-sleeved clothes made of cotton were worn, with the exception of the sampling at Tirpitzmole, where, due to the weather conditions, a rain jacket made of 100% black nylon was needed.

At each study site, one sediment sample was collected at one spot and sieved using seawater, which was pre-filtered through a 0.063-mm stainless steel sieve, into three grain size classes: 0.2–0.5 mm, 0.5–1 mm, and 1–5 mm, until a total wet weight (including size classes 0.2–5 mm) of approximately 5 kg was reached. The three grain size fractions were stored in separate glass jars, while grain sizes smaller than 0.2 mm were discarded. We used analytical sieves made from stainless steel that were stacked with the coarsest sieve on top to facilitate the process. Furthermore, the stacked sieves were mounted to a tripod to improve their handling and we placed this construction at the water line. Sediment samples were then transported to a nearby laboratory for density separation (see below). After the density separation was completed, the mineral fraction of the samples was rinsed with fresh water to remove the salt and subsequently dried for at least 72 h at 60 °C until weight consistency was achieved. To estimate the sediment content below 0.2 mm at the three sampling locations, five sediment samples with a mean (± sd) dry weight of 395 g ± 22 g were collected in December 2019 at the drift line at each site. Subsequently, a grain size analysis in ¼ phi intervals by mechanical dry sieving according to the ASTM standard (American Society of Technical Measurements) was performed for each sediment sample. This information was used to determine the total dry weights (including all size fractions) of the sediment samples that were taken for the microplastic analysis at the three sites in 2016 by calculating:

$$ x\times \left(\frac{100}{100-y}\right) $$

with *x* representing the dry mass of the sediment sample without the fraction below 0.212 mm (in gram) and *y* being the percentage share of the fraction below 0.212 mm as assessed in the grain size analysis.

Results of the grain size analysis were classified according to Folk (1954) to identify the sediment type. Statistical parameters were calculated on the basis of the grain size distribution of Wentworth (1922). Sediment classification and statistics are shown in Table 1. Grain size analysis was performed in GRADISTAT (version 8.0), a grain size distribution and statistics software for Microsoft Excel (Blott and Pye 2001). Grain size distribution plots for each sampling location are included in Online Resource 1.

**Table 1:** Sediment classification and statistics of five beach sediment samples collected at each of the three sites in the Kiel Fjord. Values are presented as mean ± standard deviation

Sample	Textural Group	(D_90_–D_10_) (μm)	Gravel (%)	Sand (%)	Mud (%)
Tirpitzmole	Slightly Gravelly Sand	524.3 ± 81.2	0.8 ± 0.6	99 ± 0.6	0.2 ± 0.0
Bülk	Sand	213.4 ± 2.2	0.0 ± 0.0	99.5 ± 0.2	0.5 ± 0.2
Falckenstein	Sand	304.3 ± 50.9	0.0 ± 0.0	99.8 ± 0.0	0.2 ± 0.0

### Density separation with calcium chloride solution

Calcium chloride (CaCl_2_) was used for the density separation in order to separate synthetic fibers and fragments from the mineral fraction of the sediment. The salt is inexpensive and environmentally friendly. We produced the salt solution by dissolving the salt in deionized water under permanent stirring at 20 °C. The density of the solution was measured with an aerometer and its initial density was 1.42 g/cm^3^. However, during sample processing, the density of the solution occasionally dropped to a minimum of 1.34 g/cm^3^, depending on the water content in the sediment samples and on sample mass. Solution density was then re-adjusted to 1.4–1.42 g/cm^3^ by adding CaCl_2_ after sample processing. Sediment samples from the different sites as well as the different size fractions from each of the sites were processed separately. For each density separation, a maximum of 1 kg of sediment was mixed with 1 l of CaCl_2_ solution in an Erlenmeyer flask. This was the maximum ratio of sediment to CaCl_2_ solution that still allowed an effective ventilation of the mix during the separation. On average, 629 (± 290) g of sediment were filled into one Erlenmeyer flask and mixed with 1 l of CaCl_2_ solution. Sediment size fractions that exceeded 1 kg in wet weight were distributed to more than one Erlenmeyer flask and mixed with 1 l of CaCl_2_ solution per flask. The resulting suspensions were ventilated for a total of 40 min, and the flasks were rotated every 10 min by 90°. This was done to ensure that all the sediment in the flasks was whirled up by the air stream. Thereby, organic particles or microplastics were separated from the heavier sediment and were brought into suspension. After 40 min, we let the suspensions rest for 15 to 18 h. After this period, the supernatants were carefully sucked into a washing bottle by the use of a vacuum pump. Subsequently, they were then filtered with a 0.2-mm polyester round filter, which was placed in a Büchner funnel that was put on top of a 5-l glass bottle. When the glass bottle was full, the filtered CaCl_2_ solution was used again for further separations. The extraction step was repeated three times according to the recommendation of Besley et al. (2016), who observed an increase in microplastic recovery with an increasing number of extractions. Finally, the filter was rinsed with 35% hydrogen peroxide and stored in a closed glass Petri dish to dissolve the organic matter that was present in the sample. After 24 h, each filter was carefully rinsed with deionized water to remove remaining organic residues and stored again in a separate glass Petri dish until inspection. The applied density separation with air-venting and rotation of the Erlenmeyer flasks as well as the treatment with hydrogen peroxide was done following the approach by Stolte et al. (2015).

### Filter analysis

The filters were inspected with a stereo microscope (M8 from Wild Heerbrugg, Heerbrugg, Switzerland) at a 6- to 50-fold magnification.

Reference photos, i.e., images of polyester and cotton fibers, human hair, and wood or shell debris from marine bivalves and gastropods that were previously taken with the same stereo microscope at different magnifications, were used for particle identification. In addition to these photos, we employed the following criteria to distinguish microplastics from other materials contained in the samples (following Norén 2007): (a) plastic particles do not exhibit organic structures in any of their parts; (b) plastic fibers are equally thick in all parts and do not taper towards the ends; (c) plastic particles are uniformly colored. We distinguished between two classes of microplastics: fragments and fibers, which were assessed separately. In this text, we summarize fibers and fragments under the generic term “particles.” However, only colored fibers were classified as either natural or synthetic based on visual criteria. For transparent, white, and black fibers, such a classification was not meaningful as we were unable to reliably distinguish these fibers from cotton or other organic materials. Additionally, white-transparent fibers were difficult to identify on the white-transparent filter mesh. Hence, the amounts of these fibers that we quantified for each grain size fraction can contain an unknown proportion of synthetic fibers. Fiber and fragment abundances per grain size class were standardized to 1 kg of total dry sediment (including all size fractions).

The differentiation between fibers and fragments was necessary to compare our results with other studies that monitored microplastic abundances in beach sediments. In some studies, fibers were listed separately (Stolte et al. 2015; Chubarenko et al. 2018), or they were completely ignored (Ory et al. 2020). We photographed the fibers and fragments with a ProgRes CF USB CCD camera (Jenoptik, Jena, Germany) that has a maximum resolution of 1360 × 1024 px. The optical system was used with the ProgRes CapturePro software, version 2.9.0.1.

Of all visually identified microplastic fragments, 39% (70 of 180 fragments) were randomly sorted out for identification with a Labram HR800 confocal Raman microscope (Horiba Jobin Yvon GmbH, Bensheim, Germany). Synthetic fibers were not examined by Raman microspectroscopy as the filter sample must be exposed to air during Raman measurement. Thus, the sample is prone to airborne fiber contamination, which we assumed is more likely to occur compared to airborne contamination with fragments > 0.2 mm. The fragments were first measured with a 532-nm wavelength laser and a 600-grooves/mm diffraction grating. Parameters like beam attenuation and acquisition time were chosen for each particle individually, depending on sensitivity to laser energy and signal-to-noise ratio of the spectra. In cases of a high fluorescence signal, we repeated the measurement with a 785-nm wavelength laser. If it was still not possible to get spectra of sufficient quality, the corresponding fragments were cut in order to obtain a smooth surface that was less affected by potential weathering effects. Spectra identification was performed with the KnowItAll spectral data base (Bio-Rad, Philadelphia, PA, USA). In case of poor signal-to-noise ratios, only the most characteristic peaks could be used for the identification procedure. Hence, it is possible that the fragments do not consist of the polymer with the best match of the database spectrum, but of a material with a similar spectrum and almost identical dominant peaks. If, for example, the classification of a noisy spectrum is high-density polyethylene (HDPE), alternative spectral matches could be low-density polyethylene (LDPE), polyethylene wax, or polyethylene glycol. Nevertheless, alternative identifications would occur for similar material classes and the attribution to polymer or non-polymer is not affected by this uncertainty.

### Assessment of background contamination

To assess a potential background contamination, i.e., fibers or fragments that do not originate from the sediment but from the laboratory equipment or the room air, two extra blank samples without sediment were processed prior to processing the sediment samples. Four liters of CaCl_2_ solution were equally distributed to four 1-l Erlenmeyer flasks, which were previously rinsed with deionized water to remove all particles from the inner walls of the flasks. Each of these solutions was then ventilated for 40 min and, during this time, the Erlenmeyer flasks were rotated by 90° every 10 min. Then, each of the solutions was vacuum-sucked into a separate pre-cleaned glass bottle with a volume of 2 l. The total 4 l of CaCl_2_ solution were then filtered through a single 0.2-mm polyester round filter. Finally, the filter was inspected under a stereo microscope with a magnification of 25 to 40. In the first of these extra blank samples, we found 11 fragments of which one was classified as plastic based on the criteria listed above. In addition to this, 83 transparent, 29 blue fibers, and one red fiber were found. Eighteen of the 29 blue fibers and the one red fiber were classified as synthetic. We then applied the following measures and changes to reduce contamination during sample processing: (a) the entire laboratory workspace and the equipment were rinsed with tap water after each work step; (b) all materials and surfaces were covered with aluminum foil during breaks in the process; (c) a lab coat made of cotton as well as latex gloves were worn during sample processing; and (d) the glass containers and Erlenmeyer flasks were permanently covered with aluminum foil. As a consequence, background contamination decreased from 19 synthetic fibers in the first extra blank sample to one fiber in the second extra blank sample, while no plastic fragments were found in the latter. Furthermore, we tried to minimize contaminations by not wearing synthetic clothes when processing the samples in the laboratory (Wesch et al. 2016).

## Results

### Sediment grain size analysis

At the sewage treatment plant, the sediment consisted mainly of medium sand (Table 1). It was classified as well sorted and showed a unimodal grain size distribution. The sediment at Falckenstein beach was similar: It was moderately well sorted, showed a unimodal grain size distribution, and consisted mainly of medium sand. We assessed the share of sediment below 0.212 mm, which was the mesh size used for the grain size analysis, as percentage of the total weight. It was 5.5% ± 0.7% (mean ± sd) at Bülk and 5.3% ± 0.3% at Falckenstein. In contrast to this, the sediment at Tirpitzmole showed a bimodal grainsize distribution and consisted of gravelly sand with two peaks: one at fine sand and one at medium sand. Here, the sediment fraction below 0.212 mm constituted 31.9% ± 2.6% of the total weight.

### Abundance of microplastic fibers and fragments in sediment samples

**Fig. 3:**
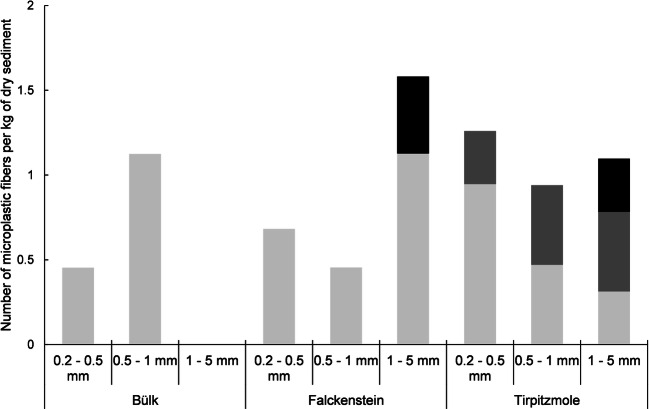
Number of variously colored microplastic fibers per grain size class in sediment samples from three locations along the western shore of the Kiel Fjord. Light gray = blue fibers, dark gray = red fibers, black = other colored fibers

**Fig. 4:**
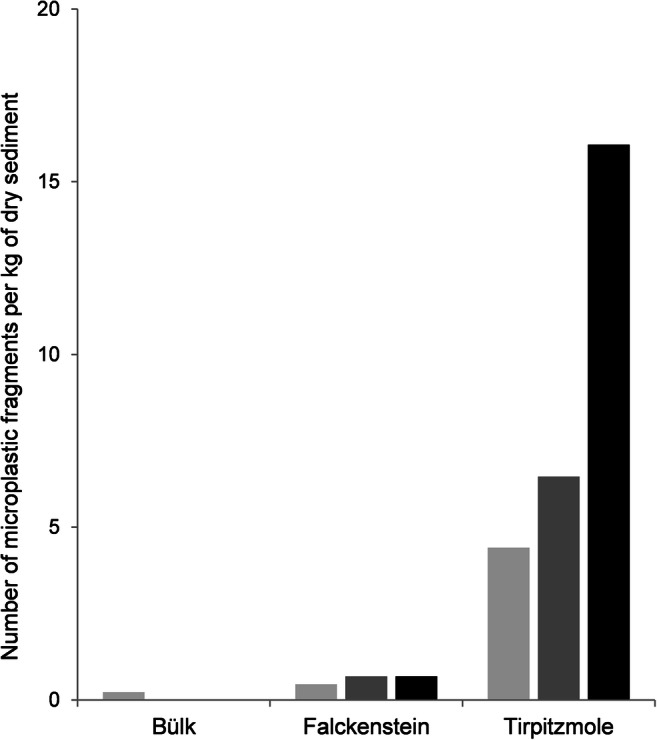
Number of microplastic fragments per grain size class in sediment samples from three locations along the western shore of the Kiel Fjord. Light gray = 0.2–0.5 mm, dark gray = 0.5–1.0 mm, black = 1.0–5.0 mm

**Fig. 5:**
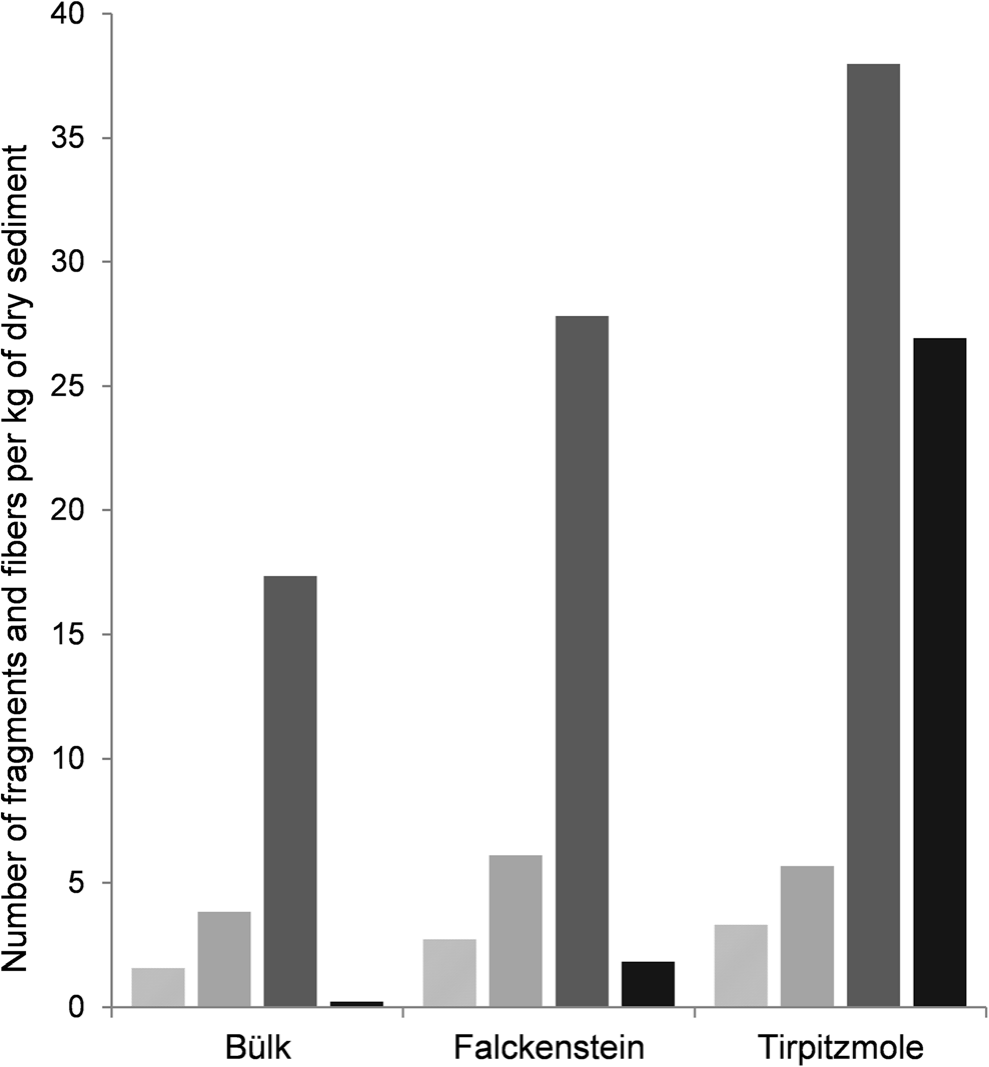
Number of synthetic fibers (light gray); colored fibers (gray); all fibers including transparent, black, and white fibers (dark gray); and plastic fragments (black) per kg of dry sediment at the sampling sites in Kiel Fjord

Blue fibers and variously colored fragments were the most abundant microplastic particles in the sediment samples from Kiel Fjord (Figs. 3 and 4). Pink, orange, and yellow fibers were observed in low numbers in the sediment samples from Falckenstein and Tirpitzmole. Red fibers were more abundant, but were only found at Tirpitzmole (Fig. 3). The total abundance of microplastic particles, i.e., synthetic fibers and fragments summed across the entire grain size range that we sampled (i.e., 0.2–5 mm), was lowest in Bülk, where 1.8 particles per kg dry sediment were found. It was slightly higher in Falckenstein with 4.5 particles per kg dry sediment, but was by one order of magnitude higher at Tirpitzmole with 30.2 particles per kg dry sediment (Fig. 4). This was primarily due to the high loads of fragments that were found there (Fig. 5).

At Falckenstein and Tirpitzmole, the abundance of microplastic particles decreased with decreasing grain size. It was the highest in the 1–5-mm size class and the lowest in the 0.2–0.5-mm class, while in Bülk no microplastics were found in the coarsest grain size class (Figs. 3 and 4).

Of the 70 fragments that were sorted out for inspection with the Raman microscope, 54 (77%) were confirmed as being microplastics. Three fragments showed a spectrum that could not be related to any polymer spectrum from the database that was used and are therefore considered non-polymers. The remaining 13 spectra (18% of all pre-sorted fragments) were affected by a high fluorescence signal at both laser wavelengths and could not be evaluated. Only four different polymer classes were found among the measured fragments: polyethylene (20 fragments), polystyrene (21 fragments), polypropylene (9 fragments), and polyamide (4 fragments). The number of examined fragments by Raman microspectroscopy for each sample is shown in Table 2 and the composition of polymers is shown in Table 3.

**Table 2:** Visually identified and pre-sorted microplastic fragments from each size class and sampling location. Microplastics were confirmed by Raman microspectroscopy

Sample site	Size class in mm	Fragments visually classified as microplastics	Sorted and examined fragments	Confirmed microplastics
Bülk	0.2–0.5	1	0	0
	0.5–1	0	0	0
	1–5	0	0	0
Falckenstein	0.2–0.5	2	0	0
	0.5–1	3	1	0
	1–5	3	1	1
Tirpitzmole	0.2–0.5	28	14	7
	0.5–1	41	17	14
	1–5	102	37	32
Total		180	70	54

**Table 3:** Polymer composition of identified microplastic fragments. Microplastics were confirmed by Raman microspectroscopy

Sampling location	Polyethylene	Polystyrene	Polypropylene	Polyamide	Not identified/no polymer	Sum
Bülk	0	0	0	0	0	0
Falckenstein	0	1	0	0	1 (no polymer)	2
Tirpitzmole	20	20	9	4	13 + 2 (no polymer)	68
	20	21	9	4	16	70

## Discussion

Microplastic fibers and fragments were found at all three sites that we sampled along the western shore of the Kiel Fjord. Interestingly, we observed a large variability in the abundance of these particles at a relatively small spatial scale (< 20 km). The highest abundance was observed at Tirpitzmole, where the share of the finest sediment fraction (< 0.2 mm) was the highest. This indicates that the conditions at this site are calm, what presumably led to the accumulation of micro- and macroplastic debris. In contrast to this, the sites Bülk and Falckenstein represent high energy environments, where fine sediments are rather exported. These sites also showed lower microplastic loads than Tirpitzmole. The assumption that the abundance of microplastics is a function of exposure to wind and waves is supported by a review by Harris (2020), who found that high microplastic abundances were commonly observed in low-energy environments that exhibit a high trapping efficiency for fine sediments. However, the exact relationship between microplastic abundance and sediment grain size is still not resolved (Graca et al. 2017). In a study from the Polish coast, no relationship between sediment characteristics and the abundance of microplastics was detected (Urban-Malinga et al. 2020) and this is in line with previous findings from other sea areas (Mathalon and Hill 2014; Alomar et al. 2016).

The rather low quantities that we observed near the sewage treatment plant in Bülk were unexpected, since these plants cannot completely retain cosmetic beads and textile fibers, which are commonly contained in household waters (Mintenig et al. 2017). Although their retention rates can reach 98%, they still release a substantial amount of microplastics into the environment due to the large volume of wastewater they process (Anderson et al. 2016). German sewage plants, for example, are supposed to release a few hundred million to billions of microplastic particles every year (Mintenig et al. 2017).

The fact that we observed the lowest microplastic abundances near the sewage plant can be explained by characteristics of the site. First, the water outlet of the sewage plant is relatively far away from the shore, since the pipeline of the plant expands about 1040 m in a north-easterly direction into Kiel Bight (Wuttke M, personal communication). Since the main wind direction in this part of the Western Baltic is south-west, particles that are released from the outlet and that are neutrally or positively buoyant should be transported away from the coastline. Swimming and water sports are not permitted near the outlet pipeline, and the surrounding beach is predominantly covered with coarse gravel and cobbles and is therefore not attractive for promenaders. As a consequence, beach use and littering are low at this site. Hence, if autochthonic macroplastic litter should be a major source for microplastics in beach sediments, what has not been confirmed by empirical data yet, the beach in Bülk should exhibit low loads of microplastics. Another possible reason for the low abundances of microplastics that we observed near the sewage treatment plant could be the size range in which we looked for synthetic particles (i.e., 0.2–5.0 mm). A study that assessed the abundance of microplastics in the purified wastewater of a treatment plant in Oldenburg, Germany, equipped with the same filter system as the plant in Bülk, found that particle abundances increased with decreasing particle size (AWI 2016). In the cited study, particles in the size range of 1.0–5.0 mm accounted for only 0.03 to 1.84% of the total plastic load in the water after the final purification step, while particles in the size range of 0.025–1.0 mm accounted for the rest. However, more than 95% of all particles that were found were smaller than 0.2 mm. Since our detection limit was 0.2 mm, it is possible that we overlooked a substantial part of the microplastics in the sediment that was taken from near the sewage treatment plant. A recent study investigated the abundance of microplastics > 0.3 mm in the surface waters of Kiel Fjord and also included the area around the outlet of the sewage plant in their sampling (Ory et al. 2020). There, the authors found abundances of these particles that were lower than in other parts of the fjord. They explained this by the effective filter cloth with a pore size of 0.04–0.06 mm that is used as the last filtration step in the sewage processing (Ory et al. 2020).

The number of microplastics found at the beach in Falckenstein was slightly higher than near the sewage treatment plant. Since this place is heavily used by locals and by tourists during the summer months, the level of littering here is higher than at the other two sites that we sampled. During the Coastal Cleanup Day in September 2017, 3951 litter items weighing more than 150 kg were found in Falckenstein along a beach length of 2.7 km (Bratz H, personal communication). Most common litter items were cigarette butts (1092), food wrappers (659), plastic bottles/plastic bottle caps (423), and foam/plastic pieces (342). In contrast to this, in Bülk, 400 litter items weighing 71 kg were found along a beach length of 2.3 km. Most common litter items were foam/plastic pieces (178), food wrappers (65), and cigarette butts (39) (Bratz H, personal communication). If the plastic litter is not exported by wind and wave action, but disintegrates at site, the amounts of macroplastics that are released at the beach should lead to an increased load of microplastics. When interpreting the data from a beach sampling in the Western Baltic Sea, Stolte et al. (2015) also explained the high abundance of microplastics, which were found in Warnemünde, Germany, with the intense beach use in this region.

At Tirpitzmole, a site that is located in between a naval base and a marina for recreational boats, a high quantity of plastic litter, such as bottles, packaging materials, food containers, styrofoam pieces, bags, and foils, in the size range between 5 and 500 mm was observed during the sampling (Schröder K, personal observation). The material was either deposited on the beach or it was floating in the shallow water near the drift line. Most if not all of this material was presumably imported from the fjord, because flotsam constantly accumulates in this semi-enclosed place that opens to the south-east. Large-sized plastic debris is introduced into the Kiel Fjord by littering, water sport activities, and operations in Kiel harbor, public events such as Kiel Week or by seagulls that search trash bins for food. Tirpitzmole is also rarely visited by people, since the beach area is small, not attractive for swimming as well as sunbathing, and difficult to access. Fragmentation processes that take place at this site are therefore a likely explanation for the high abundance of microplastics that we found at Tirpitzmole.

Ory et al. (2020) suggest that substantial amounts of the microplastics that can be found floating in the fjord originate from the Kiel storm drainage system that discharges runoff water from roads and pavements directly into the fjord. In this system, the water, which may transport plastic debris, is just coarsely filtered by a rake. Tirpitzmole is close (about 2 km) to the most frequented promenade in Kiel that has several storm drainage pipes that lead into the fjord. In our study, at Tirpitzmole, fragments accounted for 90% of the plastics, while fibers were less common and the total loads (30.2 microplastics per kg dry sediment) were seven times higher than in Falckenstein (4.5 microplastics per kg dry sediment) and 17 times higher than near the sewage treatment plant in Bülk (1.8 microplastics per kg dry sediment).

Our study shows that microplastics’ loads and their composition can vary widely even on small spatial scales (Tirpitzmole versus Falckenstein/Bülk), but can also be similar despite of differences in beach use and proximity to a sewage plant (Falckenstein versus Bülk).

While some studies from the Baltic Sea region found highest microplastic loads at beaches near urbanized areas (Graca et al. 2017) or at beaches with high touristic activity (Stolte et al. 2015), Hengstmann et al. (2018) found no such positive correlation between tourism/degree of urbanization and microplastics at sites in the southwestern Baltic Sea. This indicates that the abundance of microplastics in beach sediments is presumably determined by a complex interaction between, for instance, beach use, degree of urbanization, wastewater discharge, river discharge, location and exposition of the sampling site, climate conditions (e.g., wind patterns), or hydrodynamics (Browne et al. 2011; Stolte et al. 2015; Graca et al. 2017).

So far, only two further studies assessed the abundance of microplastics in beach sediments from the German Baltic Sea coast. Stolte et al. (2015) sampled drift line sediments near Rostock as well as on the islands of Rügen and Usedom. The authors employed a methodology that was similar to the one used in this study, while the sites they sampled were located 200–250 km east of the Kiel Fjord. Furthermore, the authors distinguished between fibers and fragments, which were between 0.055 and 1.0 mm in size (0.2–5.0 mm in this study). They found 1.7 ± 2.0 (mean ± sd) colored fragments per kilogram of dry sediment and 117 ± 127 fibers at the drift line, including white and transparent ones (Table 4). Although, the lower size limit considered by Stolte et al. (2015) was lower than the one assessed in this study, we observed more microplastic fragments (9.7 ± 15) per kg dry sediment (averaged across all samples and grain size classes). However, this high quantity is mainly due to the large amounts of fragments we observed in the sediment from Tirpitzmole (26.9 per kg dry sediment). If we would exclude this site from the analysis, fragment concentrations found in the Kiel Fjord would be very similar to those observed by Stolte et al. (2015). The concentration of colored synthetic fibers found in this study (2.5 ± 0.9 per kg dry sediment) was similar to those observed at the eastern German Baltic Sea coast by Stolte et al. (2015), who found 4.5 ± 3.8 colored fibers per kg dry sediment. If we would consider all colored fibers that were found in the samples from Kiel Fjord, irrespectively of whether they were visually classified as natural or synthetic, we would come to an average of 5.2 ± 1.2 fibers per kg dry sediment. This value is still close to the one observed by Stolte et al. (2015).

**Table 4:** Abundance of microplastics in different regions of the southern Baltic Sea. Microplastic abundances in this study include fragments and fibers. In the Gulf of Gdansk, three locations were sampled: a) beach dune, b) cliff during calm conditions and c) after storm

Region	Particle size	Microplastic abundance (particles/kg DW)	Source
Kiel Fjord (Germany)	0.2–5 mm	1.8 Bülk 4.5 Falckenstein 30.2 Tirpitzmole	This study
Mecklenburg- Vorpommern (Germany)	0.055–1 mm	2.0 ± 2.3 (mean ± SD) fragments 4.5 ± 3.8 (mean ± SD) fibers	Stolte et al. (2015)
Rügen (Germany)	0.063–5 mm	50.2–79.1 (mean) fibers 27.7–32.7 (mean) fragments	Hengstmann et al. (2018)
Gulf of Gdansk (Poland)	0.045–5 mm	a) 34 ± 9 particles b) 49 ± 6 particles c) 31 ± 4 particles	Graca et al. (2017)
Kaliningrad (Russia)	0.5–5 mm	1.3–36.3 (mean) particles	Esiukova (2017)
Kaliningrad (Russia)	0.5–5 mm	364.3 ± 172.6 (mean ± SD) particles 204.5 ± 154.6 (mean ± SD) fibers 135.3 ± 134.9 (mean ± SD) fragments	Chubarenko et al. (2018)
Poland	0.0027–5 mm	160 ± 86 (mean ± SD) particles	Urban-Malinga et al. (2020)
Curonian Spit (Lithuania and Russia)	0.5–5 mm	44.5 ± 52.4 (mean ± SD) particles	Esiukova et al. (2020)

Hengstmann et al. (2018) analyzed beach sediments from the high water line and from the beach plateau of the Isle of Rügen employing an elutriation column and Nile Red staining. The overflow of the elutriation process was filtered through a 0.063-mm sieve and the retained particles were subsequently dried and stained with Nile Red. At four sampling locations, Hengstmann et al. (2018) found on average between 27.7 and 32.7 fragments and 50.2 and 79.1 fibers per kg dry sediment (minimum mean value − maximum mean value). Considering the synthetic fibers found in our study (2.5 ± 0.9 per kg dry sediment), fiber abundances in the study of Hengstmann et al. (2018) were by one order of magnitude higher (Table 4). Only if we would consider all fibers (including colored, white-transparent, and black fibers) that we observed in our material (27.7 ± 10.3 per kg dry sediment), the fiber loads in Kiel Fjord beach sediments are similar to the concentrations observed by Hengstmann et al. (2018). One possible explanation for this difference is that the use of the staining method led to an overestimation of the abundances of synthetic fibers, since Nile Red is known to stain also natural materials (Shim et al. 2016; Tamminga et al. 2017). In contrast to our study, microplastic abundances in sediment samples from the Island of Rügen increased with decreasing grain size, while the lowest fiber and fragment abundances were found in the biggest size class (1–5 mm). We found the highest microplastic loads in the largest size range at two of our three sampling locations (Tirpitzmole and Falckenstein).

Graca et al. (2017) conducted a microplastics survey in the Gulf of Gdansk (Poland, 550 km east of Kiel Fjord). The authors considered a particle size range of 0.045–5 mm in sediment samples that were taken from the middle of two dune beaches and from a cliff beach. At the latter site, they also sampled before and after a storm event. For assessing microplastic abundances, the authors distinguished between fibers, fragments, and films. Their methodology was similar to the one used in this study: A NaCl-solution with a density of 1.2 g/cm^3^ was added to the wet sample; the suspension was shaken and left undisturbed for deposition. Then, the supernatant was filtered through a 0.045-mm sieve. This procedure was repeated three times. Graca et al. (2017) found 34 ± 9 microplastics, i.e., fragments, fibers, and films, per kg dry sediment at the dune beach, 49 ± 6 in sediments underneath the sand cliff during calm sea conditions and 31 ± 4 after a storm (Table 4). After pre-sorting the material visually, representative particles were identified by IR-spectroscopy. The particle concentrations found in this study were similar to the ones we observed for the Kiel Fjord, but were lower than those observed by Hengstmann et al. (2018). Urban-Malinga et al. (2020) investigated the abundance and composition of microplastics at 12 beaches along the Polish coast that differ in their degree of touristic usage and urbanization as well as with regard to sediment characteristics. In the swash zone (zone of wave action on the beach), the authors sampled the upper 5 cm of the sediment and mixed 1 dm^3^ of it into a NaCl-solution (1.2 g/cm^3^). After deposition, the supernatant was filtered (2.7 μm) and the density separation was repeated three times per sample. The dried filters were examined under a stereo microscope, and potential microplastics were grouped into fibers, fragments, granules/pellets, or films. Finally, representative particles were analyzed with IR-spectroscopy. Microplastic abundances ranged between 76 ± 7 and 295 ± 182 (mean ± standard deviation) particles per kg dry sediment, with an overall mean of 160 ± 86 per kg dry sediment. These values are substantially higher than our findings of 1.8 to 30.2 particles per kg dry sediment. Depending on the site, fibers (six study sites) or fragments (five study sites) dominated along the Polish coast, whereas in our study synthetic fibers dominated in Bülk and Falckenstein (1.6 and 2.7 per kg dry sediment, respectively) (1.6 and 2.7 per kg dry sediment, respectively), while at Tirpitzmole fragments were more abundant with 26.9 per kg dry sediment (Fig. 5).

Esiukova (2017) investigated microplastic loads (0.5–5 mm) near Kaliningrad in the south-eastern Baltic Sea, 650 km east of Kiel Fjord. Samples were taken from the upper two centimeters of the sediment in the drift line of 13 beaches, dried and sieved into two size classes (0.5–1 and 1–5 mm). Then, potential microplastics were separated from organic debris or glass fragments under a microscope, before a density separation with a ZnCl_2_-solution (density 1.6 g/cm^3^) was applied. For this purpose, 400 g of a sample were filled into a glass container and ZnCl_2_ was added. The density solution was added to the sediment sample, stirred and left for deposition. Floating particles were filtered and identified as microplastics following the criteria suggested by Norén (2007). Esiukova (2017) distinguished between industrial pellets, granules, foamed plastics, fibers, films, and fragments and found 1.3–36.3 (minimum to maximum mean value) microplastics per kg of dry sediment (Table 4). These values are similar to what we observed in the Kiel Fjord.

Chubarenko et al. (2018) examined beach sediments in the same region in the size range of 0.5–5 mm. Sediment samples at the drift line were collected applying the method from Esiukova (2017). The samples were stored in polyethylene bags and were processed according to Zobkov and Esiukova (2017), who based their approach on NOAA recommendations (Masura et al. 2015). The authors applied two density separations with a ZnCl_2_-solution (density 1.6 g/cm^3^), filtered the supernatants, and removed organic materials and calcites with hydrogen peroxide as well as HCl (described in Zobkov and Esiukova 2017). Finally, particles were identified under a stereo microscope with the help of UV-light, mechanical stretching, and a hot needle (Norén 2007). On average, the authors found 364.3 ± 172.6 (mean ± standard deviation) microplastics per kg dry sediment with a minimum of 53 and a maximum of 572 (median: 374) particles. The minimum value is comparable to what we observed at Tirpitzmole, while all other abundances were substantially higher. The authors explained the high variability in the abundances rather by oceanographic and atmospheric processes, such as wave- or storm-induced sediment transport, than by anthropogenic influences like urbanization. Additionally, they found the highest microplastic abundances in coarse sands indicating that these particles were brought there by waves, while we observed the highest abundances in fine sediments, what hints at the fragmentation of larger plastic debris at site.

Esiukova et al. (2020) sampled sediments at six locations along the Curonian Spit UNESCO National Park following the method described in Esiukova (2017). Samples were dried and sieved into four size classes (0.5, 1.0, 2.0, and 5.0 mm). Then, large particles were inspected under a stereo microscope using an UV-lamp, mechanical stretching, and a hot needle. Potential microplastics from this pre-selection were picked and identified using Raman spectroscopy. The rest of the material was processed with the modified NOAA density separation (Zobkov and Esiukova 2017). The median microplastic abundance was 30.2 ± 15.6 (0.5–5.0 mm) per kg of dry sediment (Chubarenko et al. 2020) and the overall mean abundance for the drift line was 44.5 ± 52.4 (mean ± standard deviation) with a minimum value of 5 and a maximum value of 177 plastic particles per kg dry sediment (Esiukova et al. 2020). These values are similar to the abundances observed in this study. However, the maximum abundance found by Esiukova et al. (2020) (177 particles) was considerably higher than what we found at Tirpitzmole (30.2 particles).

The results of the different studies indicate that microplastics are ubiquitous in beach sediments of the southern Baltic Sea, while the concentrations vary between single and hundreds of particles per kg sediment. However, a direct comparison between the studies is problematic, since different methods were used. In this context, synthetic fibers are most problematic, because they are commonly difficult to distinguish from natural materials (Stolte et al. 2015). Additionally, the likelihood of airborne fiber contamination is high in most laboratory environments. For this reason, fibers were assessed in different ways in previous studies. Either all fibers were visually classified as natural or synthetic (Esiukova 2017; Chubarenko et al. 2018), or just colored fibers were visually classified (Stolte et al. 2015). Furthermore, fibers were stained and visually identified under UV-light (Hengstmann et al. 2018), or they were visually pre-sorted and examined by IR- or Raman spectroscopy (Graca et al. 2017; Urban-Malinga et al. 2020; Esiukova et al. 2020). Finally, some studies completely excluded them due to the high background fiber contamination (e.g., Ory et al. 2020).

The similarity in pollution levels across large spatial scales (i.e., hundreds of kilometers) found by studies from the south-western Baltic Sea (Stolte et al. 2015; Graca et al. 2017; Esiukova 2017; Hengstmann et al. 2018; Esiukova et al. 2020) is in contrast to our observation that the abundances of microplastics can vary substantially at small spatial scales (i.e., tens of kilometers). The latter fact presumably arose from our sampling at a site that, due to local conditions, serves as a sink for plastic debris and therefore exhibits particle abundances that may not be representative for the study area. Although, we had just one sediment sample per site, it should be noted that at each beach ~ 4.3 kg of sediment (dry weight) were sieved into the three size classes. In most other studies, much lower amounts of sediment were processed: Stolte et al. (2015) had samples with a dry weight of 450 to 960 g, Graca et al. (2017) processed 150 g (wet sediment), and Chubarenko et al. (2018) examined 3 dm^3^ (wet volume). Esiukova (2017) processed samples of about 400 g and Hengstmann et al. (2018) as well as Urban-Malinga et al. (2020) used sub-samples of 50 cm^3^ for density separation. In this context, we are convinced that not only the number of replicates but also the total sample mass that is processed is important for the significance of a study.

Among the studies that so far assessed microplastics in beach sediments in the southern Baltic Sea (Stolte et al. 2015; Graca et al. 2017; Esiukova 2017; Hengstmann et al. 2018; Chubarenko et al. 2018; Esiukova et al. 2020; Urban-Malinga et al. 2020), only Graca et al. (2017), Esiukova et al. (2020), Urban-Malinga et al. (2020), and this study used spectroscopic methods to verify their visual identification. Studies that compared visual identification methods for microplastics to other techniques such as spectroscopy or gas chromatography/mass spectrometry revealed that overestimation of particle loads is a likely consequence of the visual analysis. In one case, up to 70% of the particles that were first classified as plastics (by visual methods) were later identified as non-plastic (by spectroscopy) (Hidalgo-Ruz et al. 2012). In another study, only 1.4% of the visually classified microplastics were later verified as such by Fourier transform infrared spectroscopy (FTIR) (Löder and Gerdts 2015).

However, in our study, 70 randomly chosen fragments, which were visually classified as synthetic, were analyzed with Raman spectroscopy and 77% (54 fragments) of them were then confirmed as microplastics. Only three fragments turned out to be a non-polymer, while the rest (13 fragments) could not be identified. This indicates that at least for larger fragments > 200 μm pre-selection by visual identification can lead to reliable results. This is supported by Urban-Malinga et al. (2020), who found that 91% of all particles they visually pre-sorted were identified as microplastics by IR-spectroscopy. Mintenig et al. (2020) in agreement with Koelmans et al. (2019) suggested that 75% or at least 50 visually pre-sorted particles should be analyzed by spectroscopic methods to reduce uncertainty.

The polymer composition of microplastics observed in this study (Table 3) is similar to what Ory et al. (2020) reported for surface-floating microplastics in the Kiel Fjord. They found that most microplastics were made of polyethlene (45%), followed by polypropylene (17%) and polystyrene (8%). Also, Urban-Malinga et al. (2020) found that most of the particles they analyzed with IR-spectroscopy were either polypropylene, polyethylene, or polystyrene. This picture resembles the polymer composition of packaging materials, which is the most important market segment for plastics in Europe (PlasticsEurope 2019). However, polyethylene terephthalate (PET), which is also frequently used in the packaging industry, was not found in this study. This might be due to its high density (1.29 to 1.40 g/cm^3^) (Nuelle et al. 2014), which renders it more difficult to get extracted with the applied CaCl_2_ density solution (1.34 to 1.42 g/cm^3^).

To make particle identification more reliable, we did not classify fibers that were transparent, black, or white, since they cannot easily be identified as synthetic or natural (Stolte et al. 2015). One reason for this is that hydrogen peroxide can bleach out organic materials. As a consequence, fibers and fragments that might have been recognizable as organic by their color before the oxidation treatment could not be distinguished from microplastics after it. Furthermore, white and transparent fibers were difficult to distinguish from the filter material we used.

The fact that the microplastic loads that were observed by the different researchers in the southern Baltic Sea were mostly in a similar range (Stolte et al. 2015; Graca et al. 2017; Esiukova 2017; Hengstmann et al. 2018; Esiukova et al. 2020) suggests that these results are robust and can serve as a baseline for future studies that seek to identify trends in pollution levels. Nevertheless, there is still the need for additional samplings in this region and to further improve and harmonize the methodology for microplastic monitorings in order to increase accuracy and to facilitate inter-study comparisons (Lorenz et al. 2019; Imhof et al. 2017; Rocha-Santos and Duarte 2015; Löder et al. 2015).

## Supplementary information

ESM 1 (PDF 294 kb)

## Data Availability

All data used in this study will be made publicly available on PANGAEA (https://www.pangaea.de) following acceptance.
