# Supplementary material for: Microplastic abundance in beach sediments of the Kiel Fjord, Western Baltic Sea
Source: Environ Sci Pollut Res Int. 2021 Jan 23;28(21):26515–28. doi: 10.1007/s11356-020-12220-x (PMC8159790; doi:10.1007/s11356-020-12220-x)
Supplement: Supplementary file 1 — (PDF 294 kb) [file 11356_2020_12220_MOESM1_ESM.pdf]

Microplastic abundance in beach sediments of the Kiel Fjord, Western Baltic Sea

Journal: Environmental Science and Pollution Research

Kevin Schröder [a], Elke Kossel[b]; Mark Lenz[b]

a. University of Kiel, Faculty of Agricultural and Nutritional Sciences, Olshausenstr. 40, 24098 Kiel, Germany
b. GEOMAR Helmholtz Centre for Ocean Research Kiel, Marine Ecology, Düsternbrooker Weg 20, 24105 Kiel, Germany
c. GEOMAR Helmholtz Centre for Ocean Research Kiel, Marine Biogeochemistry, Wischhofstr. 1 – 3, 24148 Kiel, Germany

Corresponding author: Mark Lenz, mlenz@geomar.de, Fon: ++49 431 600 4576, Fax: ++49 431 600 1671

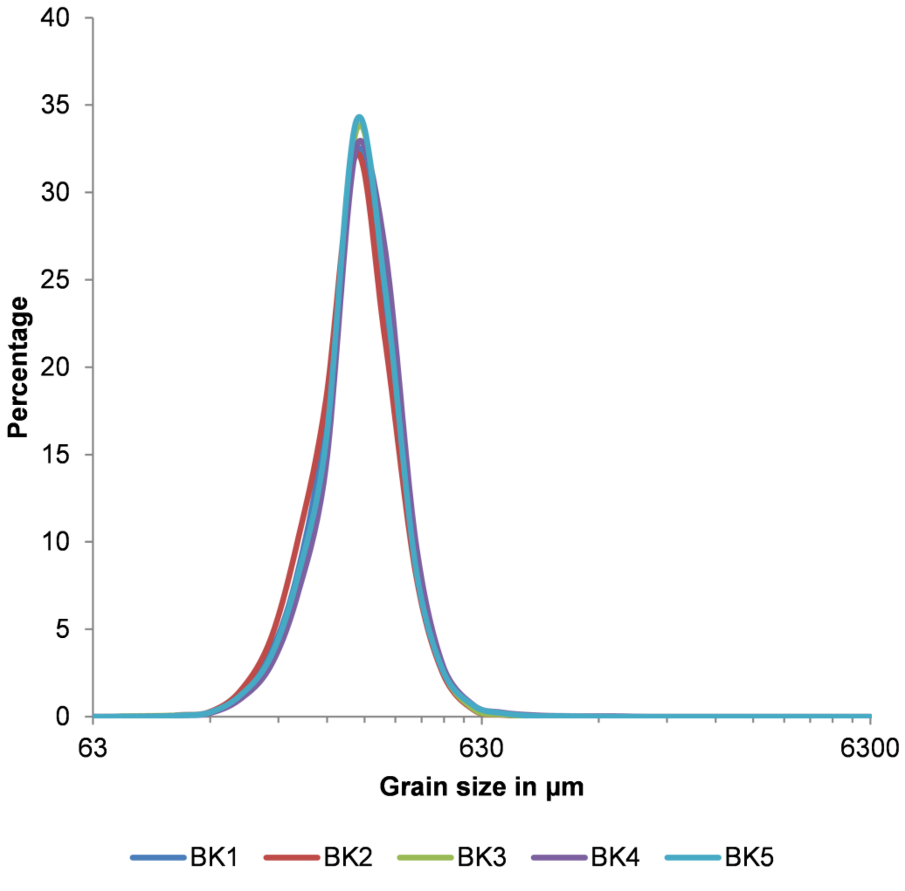

Figure 1 Grain size analysis results for five sediment samples at the sewage treatment plant Bülk (BK1 – BK5). On the x-axis the grain size is displayed on a logarithmic scale. The y-axis displays the percentage share of the whole sediment sample

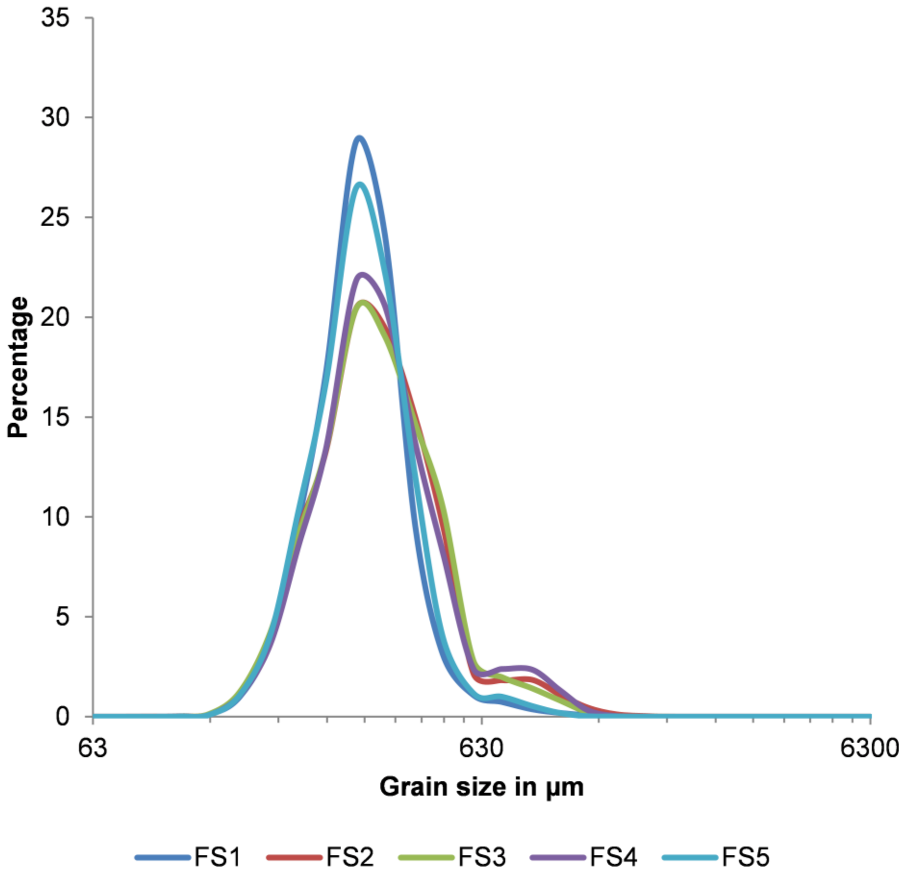

Figure 2 Grain size analysis results for five sediment samples at Falckenstein beach (FS1 – FS5). On the x-axis the grain size is displayed on a logarithmic scale. The y-axis displays the percentage share of the whole sediment sample

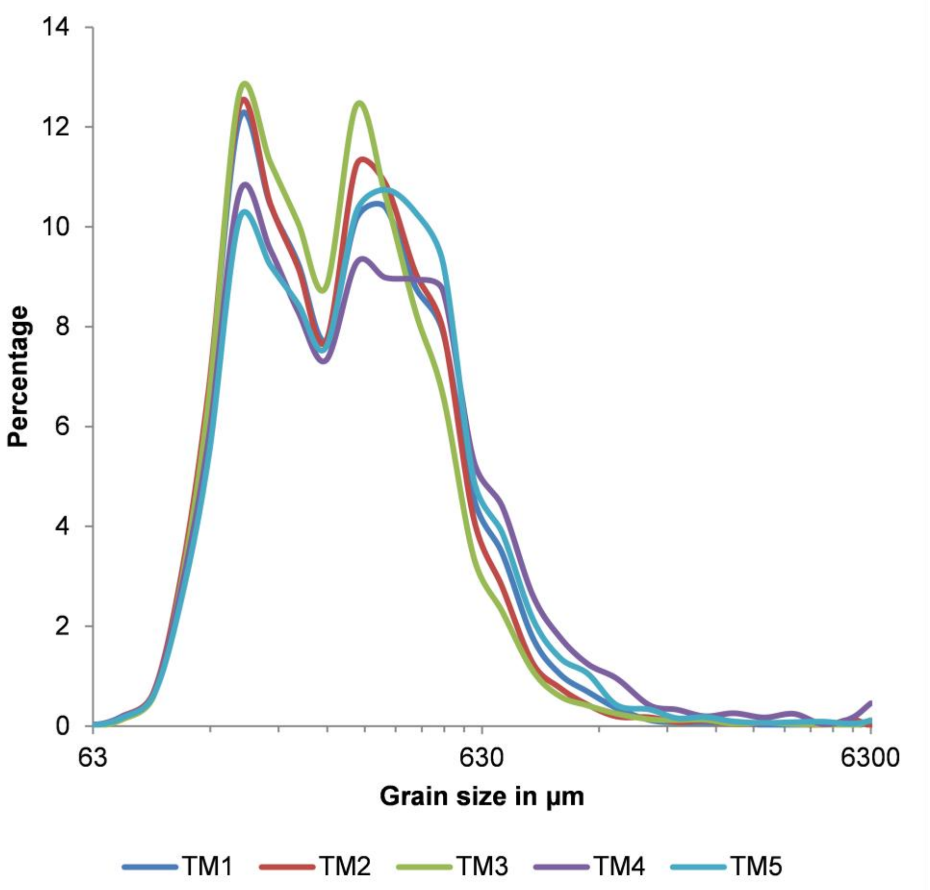

Figure 3 Grain size analysis results for five sediment samples at Tirpitzmole (TM1 –TM5). On the x-axis the grain size is displayed on a logarithmic scale. The y-axis displays the percentage share of the whole sediment sample
